# Novel Modification of the Collective Dynamic Routing Method for Sensors’ Communication in Wi-Fi Public Networks

**DOI:** 10.3390/s22228602

**Published:** 2022-11-08

**Authors:** Sergey Kozlov, Elena Spirina, Ivan Ashaev, Anna Bukharina, Artur Gaysin

**Affiliations:** Radio-Electronic and Telecommunication Systems Department, Kazan National Research Technical University Named after A. N. Tupolev-KAI, Kazan 420111, Russia

**Keywords:** IoT, energy-efficient, Wi-Fi, IEEE 802.11ax, robust algorithm, collective dynamic routing, centralized algorithm

## Abstract

The widespread use of the Internet of Things makes it relevant to use public IP networks for simultaneous access by both users and wireless sensors. To achieve this, a significant reduction in the subscriber devices’ energy consumption is required. This paper analyzes the application features of the collective dynamic routing method both with and without the use of a robust method for estimating the channel data rate for sensors’ communication in wireless public networks. Based on the analysis, a novel modification of the collective dynamic routing method has been developed that reduces the sensors’ energy consumption while keeping a high data rate and short delivery time for users. An analysis of the network load, the total data transfer rate over the network, and the parameters affecting the sensors’ energy consumption was carried out for a segment of a seamless IEEE 802.11ax network. The simulation demonstrated a high efficiency of using a novel modification of the collective dynamic routing method for access to users and wireless sensors.

## 1. Introduction

With the development of the Internet of Things, the requirements for communication networks that provide data transmission from sensors have increased dramatically. A particular challenging situation occurs with the use of wireless sensors, which have strict restrictions on energy consumption.

To date, dedicated networks based on specially developed technologies such as LoRaWAN [1], SigFox [2], Bluetooth [3], etc., or based on standards of public wireless networks, such as LTE [4], Wi-Fi [5], and 5G NR, are used to transmit data from wireless sensors. However, the use of dedicated networks leads to additional costs for the deployment, maintenance, and provision of gateways into the public network.

The use of existing public wireless networks for simultaneous servicing of both users and sensors is economically more profitable. However, the very different requirements for the data transmission volume, data rate, delays, and power consumption significantly complicate the implementation of such networks.

In modern wireless networks, high data transfer rates are provided using high-transmitting devices’ power, and small delays are due to constant listening to the medium, which requires to keep the subscriber’s device receiver constantly switched on. Both these factors lead to significant subscribers’ devices’ power consumption. It reduces their lifespan without regular recharging and complicates the use of public wireless networks for transmitting data from sensors.

Thus, it is necessary to significantly reduce the power consumption of subscribers’ devices to enable effective use of existing public wireless networks for transmitting data from sensors. The main directions in solving this problem include a reduction of the energy required for data transmission and decreasing the operating time of the subscribers’ devices’ receivers. The first task can be solved by dynamic planning of the wireless communication networks’ energy parameters [6], and the second one by improving the access to the medium methods and data flow management. Thus, in the new 3GPP specification (Revision 13) [4] and Wi-Fi standards (starting with IEEE 802.11ax) [5], changes have already been introduced to the access to the medium methods for reducing the operating time of the subscribers’ devices’ receivers. However, these changes are limited by the requirements for the maximum allowable latency to the data transmission medium for users.

Two main routing methods are used to manage data flows in modern public wireless networks: static and dynamic. However, both routing methods do not consider the intra-system interference that occurs during data transmission.

To reduce the intra-system interference in public wireless networks in the case of data transmission only by users, the authors proposed a new routing method—the collective dynamic routing (CDR) method, protected by a patent [7]. Its key features are:

- All valid routes are formed before the start of data transmission, and not during the first packet transmission.
- The optimal routes set is determined based on the criterion of minimizing the delivery time before the start of data transmission in order to reduce the intra-system interference flow.
- The packet sequence can be delivered to the same node via different routes.

However, the computational complexity of determining the optimal routes set makes it impossible to use the CDR method [7] in public wireless networks that simultaneously serve both users and sensors. Therefore, there is an urgent need to develop a novel modification of the CDR method for these network types, which is described in this article.

## 2. Related Works

Currently, specially built networks are used to ensure data transmission from wireless sensors. These networks can effectively transmit data from sensors and have low power consumption.

LoRaWAN is a low-power wireless network technology with a long-range coverage area. This technology was developed by LoRa Alliance to meet the requirements of long-range communication of wireless sensor networks with low power-consuming, low data rate communication networks. LoRaWAN cancels the need of repeaters between the nodes, thus reducing the network cost, improving the longevity of their batteries, and increasing network capacity. Altogether, this makes them suitable for wireless sensor networks (WSN) that require low energy consumption and a low data transmission rate [8,9].

SigFox was developed for the largest global IoT network to connect large numbers of objects broadcasting data. Sigfox provides a software-based communications solution, where computing is controlled in the Cloud, instead of the devices. It reduces energy consumption and connected nodes’ cost [4].

For IoT applications, Bluetooth wireless technology can be used to exchange data at short distances with minimum power consumption [3]. The use of Bluetooth in applications for sensors and controls requires not only low power consumption, but also a small amount of transmission data and infrequent communications. Bluetooth stakeholders are making great efforts to support IP so that this technology can serve IoT applications. Specification of Bluetooth 4.1 provides Bluetooth Low Energy (BLE) and IP connection to support IoT [10]. However, this mode only supports star network topologies and cannot support mesh networks.

Since dedicated networks based on specially developed technologies are designed to transfer small data volumes at low rates, it is difficult to implement the simultaneous traffic transmission from sensors and users.

An alternative option is to transfer sensor data through the existing architecture of public wireless networks of Wi-Fi [5], LTE [4], and 5G NR standards.

The first approach that allows these networks to provide wireless access to sensors is the implementation of special radio interface functions.

LTE networks support the machine-type devices’ communication (MTC), which include sensors [11]. To implement MTC in LTE, the LTE-M and NB-LTE radio interfaces are used.

In LTE-M [12], unlike users using the entire frequency resource, the sensor data transmission is carried out only over six central resource blocks, together with users. In addition, low bit rate modulation and high-redundancy channel coding are used to meet the power requirements for sensor traffic transmission. This results in the network bandwidth limitations for both sensors and users.

Additionally, for LTE technology networks, a modification of the NB–LTE radio interface [13] was proposed. In this case, a separate radio access network with its own organization of channels with a band of 200 kHz is created to transmit sensor traffic. The NB–LTE frequency channel can be integrated into the LTE or even GSM frequency grid. However, the narrow bandwidth of the channel significantly limits the maximum throughput of such networks.

The use of special radio interface functions allows public networks to provide wireless access to sensors, but significantly limits their bandwidth.

Another approach that allows public wireless networks to provide access to wireless sensors is the use of special routing methods.

One of the simplest routing methods in public networks is static routing [14]. However, this method has a number of significant disadvantages. It is necessary to manually configure routes, which is a rather difficult task for networks with a large number of nodes. Additionally, this method does not consider the influence of the changing network parameters.

To exclude these disadvantages, the dynamic routing method is used. It is implemented using a large number of protocols. Most dynamic routing protocols implement distributed routing, in which each router builds routes only for devices connected to it. For example, in LTE, the channel resources’ distribution is carried out by scheduler algorithms, considering SINR at each base station, without accounting for the channel resources’ distribution throughout the network.

Most of the dynamic routing protocols used in public networks that provide access to sensors can reduce the devices’ power consumption by jointly considering routing and network topology. One of the approaches is the self-organization of sensors in clusters. In [15], the sensors are organized into local clusters, where one sensor acts as the local base station or cluster-head. It uses localized coordination and combines data fusion into the routing protocol to reduce the amount of information that must be sent and for minimization of transmission energy. The cluster-head is selected based on the Received Signal Strength Indicator parameter. Paper [16] proposes the use of the Enhanced Received Signal Strength Indicator for choosing cluster-heads which have less fluctuation than the Received Signal Strength Indicator.

In Wi-Fi networks, a new Neighbor Awareness Network (NAN) standard [17] has been developed, which involves connecting devices to clusters. This standard uses a modified media access control to the data transmission within the cluster. This method supports the synchronous devices’ operation, which reduces the IoT devices’ power consumption [18].

However, when self-organizing sensors into clusters, it is necessary to control the network topology, which requires the service traffic transfer. It reduces the network throughput. For solving this issue, the authors of [19] proposed a joint topology control and routing protocol. It coordinates energy and routing parameters of wireless nodes based only on the traffic information.

In the existing clustering protocols, the near base station undergoes a large number of receiving, aggregating, and transmitting operations in comparison to a faraway station. This imbalance of load on the base station and lack of a structured multi-level clustering framework leads to a reduction of lifetime of WSNs. In [20], a HCR (Hierarchical Clustering and Routing) protocol is proposed to formulate a load-balanced approach for clustering while taking care of energy efficiency, reliability, and scalability.

The review in [21] shows that when using clustering, it is necessary to take into account interference both within the cluster and between network clusters. Clustering Heuristic and Channel Assignment (CHaChA), a distributed cross-layer approach for cluster formation and channel assignment that directly integrates the default IEEE 802.11 mesh protocol information and operating modes, is presented in [22]. In this paper, to avoid interference between neighboring clusters, the Airtime Link Metric is used.

To reduce the influence of interference within a cluster, routing protocols are often used, which, when building a route, consider the distribution of frequency channels.

For example, the authors of [23] propose a distributed Joint channel Assignment, Routing, and Scheduling (JARS) protocol that involves separate processing of data packets and service broadcast packets. For each packet type, the data transmission order is jointly determined, the communication channels used for transmission are selected, and data transmission routes are constructed in the cluster.

In [24], a protocol was proposed that jointly considers routing and frequency channels’ allocation based on SINR analysis. It allows to increase network throughput by using channels with a minimum interference level for data transmission.

To increase the throughput of IEEE 802.11 networks and reduce the packet transmission end-to-end delay, the authors of [25] propose a Joint Channel Assignment and Routing (J-CAR) protocol that performs local selection of a communication channel using the Channel Interference Index metric. This index is proportional to the channel utilization factor considering signal attenuation.

In [26], the Joint Routing and Channel Assignment Protocol (JRCAP) is proposed. It performs route construction together with the choice of a communication channel for IEEE 802.11 networks. It includes clustering and channel selection algorithms within a cluster using the Maximum Residual Capacity (MRC) metric, which depends on the data rate, channel frequency spacing, and channel load.

The authors of [27] consider a cross-layer QoS-aware routing protocol for real-time multimedia traffic transmission, operating at the physical and data link layers of the OSI model, for the IEEE 802.11 standard. It involves dynamically changing route metrics based on monitoring available frequency bands and allocating communication channels at the physical layer using a modified OLSR protocol.

In [28], the authors derived a distributed algorithm for joint dynamic routing and resource units’ allocation for multi-hop IEEE 802.11ax networks. This algorithm provides routing, distribution of resource units, modulation, and coding schemes’ adaptation in the network, considering mutual interference. However, the main part of this algorithm is aimed at the resource units’ allocation.

There are also other options. For example, in [29], a protocol was proposed that builds a multi-path route together with data rate adaptation. This protocol performs routing and channel data rate selection that minimizes the probability of error, both due to the effects of interference and due to communication channels’ overloading.

However, the protocols considered in [15,16,19,20,21,22,23,24,25,26,27,28,29] are used only in distributed networks with mesh topology and are not applicable to networks with a centralized structure. These protocols result in a significant increase in the load and power consumption for the nodes that are the head of the clusters. One of the approaches to solve this problem is the method presented in [30]. It is proposed to choose routes considering the battery level. Additionally, the protocols [23,24,25,26,27,28,29], which take into account the interference influence, work only within the cluster. Another disadvantage of these protocols is that all packets during a communication session are transmitted along the same routes without considering the random traffic structure.

Unlike the considered distributed routing protocols, with centralized routing [31,32,33], channel resources are distributed throughout the entire network. These routing protocols could find the more effective routes and improve the resource allocation over the network. However, these centralized routing methods consider scenarios only for sensor networks.

The protocols considered above use the dynamic and static routing methods and do not take into account the data flow’s random structure, and consequently, the random intra-system interference distribution during the simultaneous transmission of these flows. This issue is especially relevant for cases where a public wireless network is used both for transmitting data from sensors and mobile users.

The intra-system interference influence is taken into account by the CDR method presented in [7]. The effectiveness of using the CDR method in public IEEE 802.11ax networks in the case of data transmission only by mobile users was shown in [34]. However, connecting a large number of sensors to these networks leads to an exponential increase in the computational complexity of determining the optimal routes set, which makes it important to develop a new modification of this method.

## 3. The Collective Dynamic Routing Method Application for Access to Wireless Sensors

**Figure 1:**
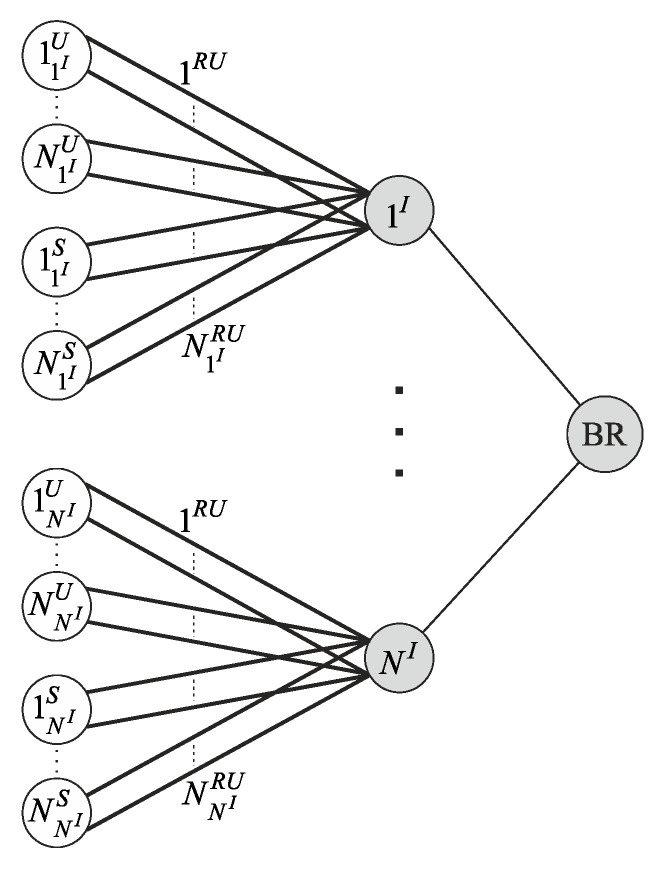
An arbitrary wireless network segment diagram.

This paper discusses an arbitrary wireless network segment. The network consists of a backbone router (BR), $N^{I}$ access points with numbers $n^{I}=\bar{1,N^{I}}$, $N^{U}$ users, and $N^{S}$ sensors (Figure 1).

Considering $N_{n^{I}}^{U}$ users and $N_{n^{I}}^{S}$ sensors connected to the $n^{I}$-th access point, then the total user and sensor numbers in the network segment are:

(1) $$N^{U}=\overset{N^{I}}{\underset{n^{I}=1}{\sum }}N_{n^{I}}^{U},$$

(2) $$N^{S}=\overset{N^{I}}{\underset{n^{I}=1}{\sum }}N_{n^{I}}^{S}.$$

The network’s task is to transfer the data vector ${\vec{d}}^{\mathrm{DL}}=\left(d_{1_{1^{I}}^{U}}^{\mathrm{DL}},\ldots ,d_{N_{1^{I}}^{U}}^{\mathrm{DL}},d_{1_{1^{I}}^{S}}^{\mathrm{DL}},\ldots ,d_{N_{1^{I}}^{S}}^{\mathrm{DL}},\ldots ,d_{1_{N^{I}}^{U}}^{\mathrm{DL}},\ldots ,d_{N_{N^{I}}^{U}}^{\mathrm{DL}},d_{1_{N^{I}}^{S}}^{\mathrm{DL}},\ldots ,d_{N_{N^{I}}^{S}}^{\mathrm{DL}}\right)$ in downlink (*DL*) and ${\vec{d}}^{\mathrm{UL}}=\left(d_{1_{1^{I}}^{U}}^{\mathrm{UL}},\ldots ,d_{N_{1^{I}}^{U}}^{\mathrm{UL}},d_{1_{1^{I}}^{S}}^{\mathrm{UL}},\ldots ,d_{N_{1^{I}}^{S}}^{\mathrm{UL}},\ldots ,d_{1_{N^{I}}^{U}}^{\mathrm{UL}},\ldots ,d_{N_{N^{I}}^{U}}^{\mathrm{UL}},d_{1_{N^{I}}^{S}}^{\mathrm{UL}},\ldots ,d_{N_{N^{I}}^{S}}^{\mathrm{UL}}\right)$ in uplink (*UL*) accumulated during the interval $T^{I}$. In this case, BR forms the data volume vector ${\vec{I}}^{\mathrm{DL}}=\left(I_{1_{1^{I}}^{U}}^{\mathrm{DL}},\ldots ,I_{N_{1^{I}}^{U}}^{\mathrm{DL}},I_{1_{1^{I}}^{S}}^{\mathrm{DL}},\ldots ,I_{N_{1^{I}}^{S}}^{\mathrm{DL}},\ldots ,I_{1_{N^{I}}^{U}}^{\mathrm{DL}},\ldots ,I_{N_{N^{I}}^{U}}^{\mathrm{DL}},I_{1_{N^{I}}^{S}}^{\mathrm{DL}},\ldots ,I_{N_{N^{I}}^{S}}^{\mathrm{DL}}\right)$ transmitted to users and sensors, and at the same time, users and sensors form the data volume vector ${\vec{I}}^{\mathrm{UL}}=\left(I_{1_{1^{I}}^{U}}^{\mathrm{UL}},\ldots ,I_{N_{1^{I}}^{U}}^{\mathrm{UL}},I_{1_{1^{I}}^{S}}^{\mathrm{UL}},\ldots ,I_{N_{1^{I}}^{S}}^{\mathrm{UL}},\ldots ,I_{1_{N^{I}}^{U}}^{\mathrm{UL}},\ldots ,I_{N_{N^{I}}^{U}}^{\mathrm{UL}},I_{1_{N^{I}}^{S}}^{\mathrm{UL}},\ldots ,I_{N_{N^{I}}^{S}}^{\mathrm{UL}}\right)$ to transfer information to the BR.

The CDR method contains two stages: the analysis stage and the routing stage. At the analysis stage, the valid routes set {w} is formed. The routing stage presents a choice of optimal routes set, ${\vec{N}}^{w\_\mathrm{opt}}$, from {w}. The optimal routes set ${\vec{N}}^{w\_\mathrm{opt}}$ is determined by the criterion of minimizing the data delivery time based on the incoming information about current volumes of delivered data, ${\vec{I}}^{\mathrm{DL}}$ and ${\vec{I}}^{\mathrm{UL}}$.

### 3.1. Analysis Stage

At this stage, the valid routes set {w} is determined. It contains the one-dimensional and the multi-dimensional routes. The formation of the valid routes set {w} is performed only during the network initial planning or configuration changing. Therefore, this task may be solved in non-real time.

All data transmitting channels can be split into dependent and independent according to their mutual influence. In the dependent channels, data transmission leads to the intra-system interference, affecting other channels. Data transmission over independent channels does not lead to intra-system interference.

When data transmission in downlink and uplink channels is going in separated periods (Wi-Fi, LTE TDD) or different frequency bands (LTE FDD), then channels can be considered as independent. It allows to split the valid routes set {w} into two disjointed subsets: $\left\{w^{\mathrm{DL}}\right\}$ contained routes that deliver data from BR to users and sensors and $\left\{w^{\mathrm{UL}}\right\}$ contained routes that deliver data from users and sensors to BR.

The route channels from $\left\{w^{\mathrm{DL}}\right\}$ and $\left\{w^{\mathrm{UL}}\right\}$ in the modern wireless communication system have mutual dependence. Therefore, the signal interference environment in these channels depends on the chosen data delivery routes. It allows to consider the intra-system interference in dependent channels by centralized routing.

To account for the intra-system interference flow that occurs during data transmission over dependent channels, end-to-end routes are used. Each end-to-end route connects users and sensors with BR and includes all channels involved in data transmission.

A one-dimensional route is an end-to-end route connecting one user or sensor with BR. The route construction is performed according to the network topology described by the network’s graph (the example is shown in Figure 1). From the graph theory, the network is represented by an undirected graph. All network devices are the graph nodes, and the channels between them are the graph edges [35]. Thus, the one-dimensional route’s construction hs the problem of finding all simple paths along the graph. One of the methods for solving this problem is graph traversal.

For graph traversal, uninformed search methods can be used: Breadth-First Search (BFS) and Depth-First Search (DFS).

According to [36], the construction of the empty list of visited nodes and the empty queue of unvisited nodes is necessary for BFS. The start node (BR) is placed at the queue’s beginning.

Graph traversal occurs by sequentially processing all nodes in the unvisited nodes queue. In the beginning, the first node is selected from the queue and placed in the visited nodes list. Next, all neighbor nodes for the selected one are found. If the found nodes are not included in the visited nodes list and are not users or sensors, then they are appended to the unvisited nodes queue and temporary routes between BR and these nodes are formed. If the found nodes are users or sensors, then they are not appended to the unvisited nodes queue. In this case, two one-dimensional routes are formed based on the available temporal routes: one route is appended to the subset $\left\{w^{\mathrm{DL}}\right\}$ and one route is appended to the subset $\left\{w^{\mathrm{UL}}\right\}$. Then, the search is repeated until the queue of unvisited nodes contains at least one node.

The main BFS method’s disadvantage is the need to form temporary routes, which increases the computational complexity of the formed one-dimensional routes.

To initialize the graph traversal using the DFS method, it is necessary to create a stack containing the start node (BR) [36].

The search for each node from the stack is performed sequentially for the next node connected to the selected one. If no such node is found, the selected node is removed from the stack. Otherwise, if the found node is not a user or a sensor, then it is appended to the stack. If the found node is a user or a sensor, two one-dimensional routes are formed based on the information available in the stack: one route is appended to the subset $\left\{w^{\mathrm{DL}}\right\}$ for downlink, and a route is appended to the subset $\left\{w^{\mathrm{UL}}\right\}$ for uplink. The search is repeated as long as there is at least one node in the stack.

In this case, using the DFS method allows to avoid construction of the temporary routes and reduce the forming time of one-dimensional routes by 33%.

Since the CDR method considers the influence of channels on each other, after finding all one-dimensional routes, it is necessary to form multi-dimensional routes consisting of one-dimensional routes that do not have common channels. In graph theory, such one-dimensional routes are called edge-disjoint [37]. This issue is similar to the one of finding maximal independent sets in graph theory [38], however, a one-dimensional routes set can be considered instead of a graph nodes set. The end solutions to this issue are valid multi-dimensional routes.

Finding multi-dimensional routes of maximum dimension could be performed using the Bron–Kerbosch algorithm [39]. Multi-dimensional routes of smaller dimensions could be obtained based on the intermediate results of the Bron–Kerbosh algorithm.

Each subset, $\left\{w^{\mathrm{DL}}\right\}$ and $\left\{w^{\mathrm{UL}}\right\}$, is the joining of the one-dimensional routes and constructed multi-dimensional routes.

Each valid route, $w_{g}$, in $\left\{w^{\mathrm{DL}}\right\}$ and $\left\{w^{\mathrm{UL}}\right\}$ contains all channels used for the data delivery, and the signal interference environment could be defined. Such approach allows to determine the channel data rates for all channels included in the route $w_{g}$ at the analysis stage. In this case, the channel data rate $V_{{\mathrm{gn}}_{n^{I}}^{U}\left(n_{n^{I}}^{S}\right)}^{\mathrm{DL}\left(\mathrm{UL}\right)}$ to (from) the user $n_{n^{I}}^{U}$ or the sensor $n_{n^{I}}^{S}$ along the route $w_{g}$ is defined as the minimum channel data rate value for all channels involved in data transmission.

With centralized access to the data transmission medium, the transmission time of each data block is known. This allows to consider the data transmission process over the network synchronous with the frame duration, $T^{F}$. The $T^{F}$ is the smallest common multiple of the data delivery intervals through all end-to-end routes. $T^{F}$ is calculated considering the physical and data link layers, as well as delays in access to the data transmission medium.

In this case, for each valid route from the subset $\left\{w^{\mathrm{DL}}\right\}$ or $\left\{w^{\mathrm{UL}}\right\}$ based on the determined channel data rate $V_{{\mathrm{gn}}_{n^{I}}^{U}\left(n_{n^{I}}^{S}\right)}^{\mathrm{DL}\left(\mathrm{UL}\right)}$, the volumes of data being delivered, ${\tilde{I}}_{{\mathrm{gn}}_{n^{I}}^{U}\left(n_{n^{I}}^{S}\right)}^{\mathrm{DL}\left(\mathrm{UL}\right)}$, to (from) the user $n_{n^{I}}^{U}$ or sensor $n_{n^{I}}^{S}$ for the frame duration $T^{F}$ can be calculated:

(3) $${\tilde{I}}_{{\mathrm{gn}}_{n^{I}}^{U}\left(n_{n^{I}}^{S}\right)}^{\mathrm{DL}\left(\mathrm{UL}\right)}=\overset{N_{g}^{M}}{\underset{n_{g}^{M}=1}{\sum }}\left(T^{F}-\tau \right)\cdot V_{{\mathrm{gn}}_{n^{I}}^{U}\left(n_{n^{I}}^{S}\right)}^{\mathrm{DL}\left(\mathrm{UL}\right)},$$

where τ is the access procedure delay, and $n_{g}^{M}=\bar{1,N_{g}^{M}}$ is the one-dimensional route number in the multi-dimensional route.

### 3.2. Routing Stage

The task of the routing stage is to determine the optimal routes set ${\vec{N}}^{w\_\mathrm{opt}\_\mathrm{DL}}$ from the subset $\left\{w^{\mathrm{DL}}\right\}$ or ${\vec{N}}^{w\_\mathrm{opt}\_\mathrm{UL}}$ from the subset $\left\{w^{\mathrm{UL}}\right\}$ according to the criterion of minimizing the data delivery time based on the incoming current volumes of delivered data ${\vec{I}}^{\mathrm{DL}}$ and ${\vec{I}}^{\mathrm{UL}}$ accumulated over the interval $T^{I}$.

In each frame with duration $T^{F}$, only one route from the subset $\left\{w^{\mathrm{DL}\left(\mathrm{UL}\right)}\right\}$ could be used. The forming of the optimal routes set ${\vec{N}}^{w\_\mathrm{opt}\_\mathrm{DL}\left(\mathrm{UL}\right)}$ is carried out in real time for the $T^{I}$ interval.

To transmit the current volumes of delivered data, ${\vec{I}}^{\mathrm{DL}\left(\mathrm{UL}\right)}$, any routes included in the subset $\left\{w^{\mathrm{DL}\left(\mathrm{UL}\right)}\right\}$ could be used. Each of the routes could be used in $N_{g}^{w\_\mathrm{DL}\left(\mathrm{UL}\right)}$ frames. Let us denote ${\vec{N}}^{w\_\mathrm{DL}\left(\mathrm{UL}\right)}=\left(N_{1}^{w\_\mathrm{DL}\left(\mathrm{UL}\right)},N_{2}^{w\_\mathrm{DL}\left(\mathrm{UL}\right)},\cdots ,N_{G}^{w\_\mathrm{DL}\left(\mathrm{UL}\right)}\right)$ as the used routes set. Then, the total data volume ${\overset{˘}{I}}_{n_{n^{I}}^{U}\left(n_{n^{I}}^{S}\right)}^{\mathrm{DL}\left(\mathrm{UL}\right)}\left({\vec{N}}^{w\_\mathrm{DL}\left(\mathrm{UL}\right)}\right)$ delivered to (from) the $n_{n^{I}}^{U}$-th user or the $n_{n^{I}}^{S}$-th sensor using a routes set ${\vec{N}}^{w\_\mathrm{DL}\left(\mathrm{UL}\right)}$ is calculated as:

(4) $${\overset{˘}{I}}_{n_{n^{I}}^{U}\left(n_{n^{I}}^{S}\right)}^{\mathrm{DL}\left(\mathrm{UL}\right)}\left({\vec{N}}^{w\_\mathrm{DL}\left(\mathrm{UL}\right)}\right)=\overset{G}{\underset{g=1}{\sum }}N_{g}^{w\_\mathrm{DL}\left(\mathrm{UL}\right)}\cdot {\tilde{I}}_{{\mathrm{gn}}_{n^{I}}^{U}\left(n_{n^{I}}^{S}\right)}^{\mathrm{DL}\left(\mathrm{UL}\right)}.$$

The data delivery time $\tau \left({\vec{N}}^{w\_\mathrm{DL}},{\vec{N}}^{w\_\mathrm{UL}}\right).$ is:

(5) $$\tau \left({\vec{N}}^{w\_\mathrm{DL}},{\vec{N}}^{w\_\mathrm{UL}}\right)=T^{F}\cdot (\overset{G}{\underset{g=1}{\sum }}N_{g}^{w\_\mathrm{DL}}+\overset{G}{\underset{g=1}{\sum }}N_{g}^{w\_\mathrm{UL}}).$$

Based on the current volumes of delivered data, ${\vec{I}}^{\mathrm{DL}\left(\mathrm{UL}\right)}$, according to the minimizing of the data delivery time criterion at the routing stage according to [40], the optimal routes set $\left({\vec{N}}^{w\_\mathrm{opt}\_\mathrm{DL}},{\vec{N}}^{w\_\mathrm{opt}\_\mathrm{UL}}\right)$ is determined as:

(6) $$\left\{ \begin{array}{l} \left({\vec{N}}^{w\_opt\_DL},{\vec{N}}^{w\_opt\_UL}\right)=\arg\underset{\left({\vec{N}}^{w\_DL},{\vec{N}}^{w\_UL}\right)}{\min}\left(\sum_{g=1}^{G}N_{g}^{w\_DL}+\sum_{g=1}^{G}N_{g}^{w\_UL}\right)\\ \begin{array}{cc} \begin{array}{l} \sum_{g=1}^{G}N_{g}^{w\_DL\left(UL\right)}\cdot {\tilde{I}}_{gn_{n^{I}}^{U}}^{DL\left(UL\right)}\geq I_{n_{n^{I}}^{U}}^{DL\left(UL\right)},\;\;n_{n^{I}}^{U}=\bar{1,N_{n^{I}}^{U}}\\ \sum_{g=1}^{G}N_{g}^{w\_DL\left(UL\right)}\cdot {\tilde{I}}_{gn_{n^{I}}^{S}}^{DL\left(UL\right)}\geq I_{n_{n^{I}}^{S}}^{DL\left(UL\right)},\;\;n_{n^{I}}^{S}=\bar{1,N_{n^{I}}^{S}} \end{array} & ,\;n_{I}=\bar{1,N_{I}} \end{array}\\ \begin{array}{c} N_{g}^{w\_DL\left(UL\right)}\geq 0\\ N_{g}^{w\_DL\left(UL\right)}\in Z \end{array},\;\;\;g=\bar{1,G} \end{array}.\right.$$

From the mathematics point of view, this issue belongs to the problems of integer linear programming (ILP). The ILP problem solution is carried out using precise methods, for example, the method of branches and boundaries, Gomori’s algorithm, etc. [41]. These methods have high computational complexity with a large dimension of input data [42].

One of the ways to reduce the computational complexity solving the ILP problem is to remove the restriction on the integers by rounding the resulting values up. In this case, the simplex method [41] can be used to obtain the optimal routes set [42].

Another option for reducing computational complexity solving ILP problems is the use of various metaheuristic approaches that differ in the generating solutions rule. This approach’s implementation assumes two options: generating possible solutions and choosing the best one among them [43], or sequential (recurrent) application of generated solutions based on the data available at the decision time doing without considering possible alternatives [41]. In [42], it was shown that for long frame durations, using recurrent algorithms allows not only to reduce computational complexity but also the data delivery time compared to the simplex method, with rounding values up.

**Figure 2:**
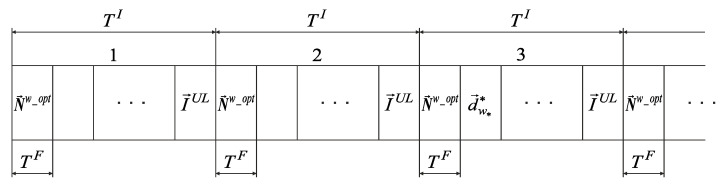
The data transmission structure in the CDR method.

Since data are transmitted for each interval in both directions (downlink and uplink), optimal sets of routes ${\vec{N}}^{w\_\mathrm{opt}\_\mathrm{DL}}$ and ${\vec{N}}^{w\_\mathrm{opt}\_\mathrm{UL}}$ may be combined in ${\vec{N}}^{w\_\mathrm{opt}}$. In this case, the data transmission structure over the network in the CDR method is shown in Figure 2.

The optimal routes set ${\vec{N}}^{w\_\mathrm{opt}}$ in the CDR method is formed by BR based on the current volumes of delivered data ${\vec{I}}^{\mathrm{DL}}$ and ${\vec{I}}^{\mathrm{UL}}$. Therefore, at each interval $T^{I}$, the data accumulation from users, sensors, and BR is performed. Since by the beginning of the first interval $T^{I}$, BR does not have information about the data volume, ${\vec{I}}^{\mathrm{DL}}$ and ${\vec{I}}^{\mathrm{UL}}$, BR sends a vector ${\vec{N}}^{w\_\mathrm{opt}}$ to all access points in the first frame $T^{F}$ of this interval, providing only for transmission ${\vec{I}}^{\mathrm{UL}}$ on the last frame $T^{F}$. Starting from the second interval $T^{I}$, the optimal routes set ${\vec{N}}^{w\_\mathrm{opt}}$ is calculated on BR according to the vectors ${\vec{I}}^{\mathrm{DL}}$ and ${\vec{I}}^{\mathrm{UL}}$. Since by the beginning of the second interval $T^{I}$ BR has not yet calculated ${\vec{N}}^{w\_\mathrm{opt}}$, in the first frame $T^{F}$ of this interval $T^{I}$, BR sends a vector ${\vec{N}}^{w\_\mathrm{opt}}$ to all access points, providing only transmission ${\vec{I}}^{\mathrm{UL}}$ on the last frame $T^{F}$. Starting from the third interval $T^{I}$, BR sends to all access points the optimal routes set ${\vec{N}}^{w\_\mathrm{opt}}$ in the first frame $T^{F}$, calculated in the previous interval $T^{I}$. The accumulated data, ${\vec{d}}_{w_{*}}^{*}$, are transmitted in subsequent frames according to the optimal routes set ${\vec{N}}^{w\_\mathrm{opt}}$.

Modern wireless networks (LTE, Wi-Fi (beginning with IEEE 802.11ax), 5G) use OFDMA technology, where data transmission is carried out using resource blocks (units) (*RU*). In the CDR method, each *RU* is considered as a separate channel. In this case, the principle of constructing multi-dimensional routes used in the CDR method leads to a sharp increase in the set’s dimensionality.

In this regard, in [44], a robust method for estimating the channel data rate for the implementation of the CDR method in seamless IEEE 802.11ax networks was developed. This approach significantly reduces the valid routes set’s dimension with a slight deterioration in its effectiveness.

### 3.3. A Robust Method for Estimating the Channel Data Rate

This paper considers a case in which downlink and uplink channels are independent. Due to various delays in the radio waves’ propagation, the delay of signals from different access points may exceed the cyclic prefix duration. According to this, intra-system interference in the downlink can be created only by signals from different access points. Therefore, it is necessary to know the *RU*’s distribution of all access points to estimate the channel data rate, considering the intra-system interference influence in multi-dimensional routes in the subset $\left\{w^{\mathrm{DL}}\right\}$. In this case, the subset includes multi-dimensional routes $\left\{w^{\mathrm{DL}}\right\}$ formed by combining all possible combinations of one-dimensional routes for all access points. Such routes’ quantity has an exponential dependence on the access point’s quantity.

**Figure 3:**
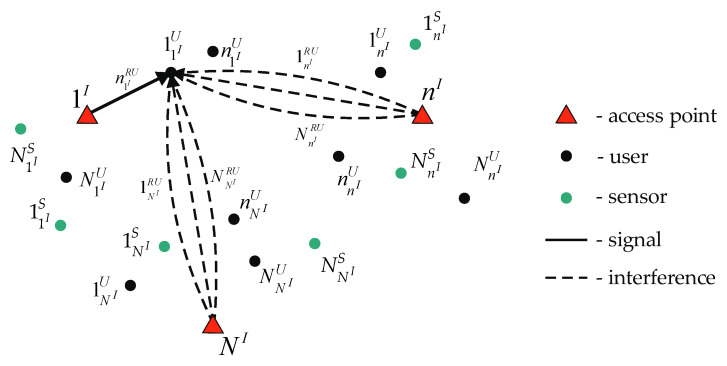
Forming a route in the downlink according to the robust method of estimating the channel data rate.

To reduce the multi-dimensional routes’ quantity in this subset, the worst-case scenario can be considered. In this scenario, each access point uses a route for data transmission that simultaneously uses all *RU*s. Figure 3 shows the data transmission case from the access point $1^{I}$ to a user $1_{1^{I}}^{U}$ using a *RU* $n_{1^{I}}^{\mathrm{RU}}$. In this case, according to the robust method, a route is used in which all other access points, $n^{I}=\bar{2,N^{I}}$, are transmitting data using all $N_{n^{I}}^{\mathrm{RU}}$ *RU*s simultaneously with data transmission from the access point $1^{I}$ to the user $1_{1^{I}}^{U}$.

In this case, the intra-system interference generated by access points will always be maximum. Therefore, the choice of a multidimensional route on one access point will not affect other access points’ operation. Then, an arbitrary wireless network segment, shown in Figure 1, can be split on separate parts containing one access point each. For each access point, the subset $\left\{w_{n^{I}}^{\mathrm{DL}}\right\}$ is created. The subset $\left\{w_{n^{I}}^{\mathrm{DL}}\right\}$ contains $N_{n^{I}}^{\mathrm{RU}}\cdot \left(N_{n^{I}}^{U}+N_{n^{I}}^{S}\right)$ one-dimensional routes and multi-dimensional routes formed on basis one-dimensional routes.

In the uplink, intra-system interference is created only by signals from different users (sensors). Therefore, it is necessary to know the *RU*’s distribution between all network users (sensors) to estimate the channel data rate, considering the intra-system interference influence along multi-dimensional routes in the subset $\left\{w^{\mathrm{UL}}\right\}$. In this case, this subset includes multi-dimensional routes formed by combining all possible combinations of one-dimensional routes for all devices. Such routes’ quantity also has an exponential dependence on the subscriber devices’ quantity.

To reduce the multi-dimensional routes’ quantity in this subset, the worst-case scenario is considered. In this case, intra-system interference is created by only one user or sensor connected for each access point. This device provides the maximum interference level for the analyzed access point. At the same time, such devices connected to the analyzed access point use all *RU*s except the *RU* used for data transmission by the considered device. Additionally, all devices connected to other access points use all available *RU*s.

**Figure 4:**
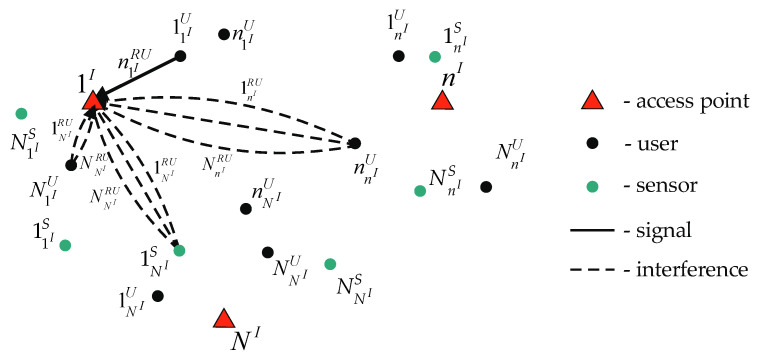
Forming a route in the uplink according to the robust method of estimating the channel data rate.

Figure 4 shows the data transmission from the case of a user $1_{1^{I}}^{U}$ to an access point $1^{I}$ using a *RU* $n_{1^{I}}^{\mathrm{RU}}$. Assume that the maximum interference to the access point $1^{I}$ is provided by users $n_{n^{I}}^{U}$ and $N_{1^{I}}^{U}$ and sensor $1_{N^{I}}^{S}$. In this case, according to the robust method, a route for data transmission is used, where the user $N_{1^{I}}^{U}$ transmits data to the access point $1^{I}$ using *RU*s from $1_{1^{I}}^{\mathrm{RU}}$ to ${\left(n-1\right)}_{1^{I}}^{\mathrm{RU}}$ and from ${\left(n+1\right)}_{1^{I}}^{\mathrm{RU}}$ to $N_{1^{I}}^{\mathrm{RU}}$ simultaneously with the data transmission from the user $1_{1^{I}}^{U}$ to the access point $1^{I}$ through the *RU* $n_{1^{I}}^{\mathrm{RU}}$. Additionally, at the same time, the user $n_{n^{I}}^{U}$ and sensor $1_{N^{I}}^{S}$ transmit data using all $N_{n^{I}}^{\mathrm{RU}}$ and $N_{N^{I}}^{\mathrm{RU}}$ *RU*s, respectively.

In this case, the choice of a multi-dimensional route on the remaining access points does not affect the operation of this access point’s devices. Then, a wireless network segment, shown in Figure 1, can also be split on separate parts containing one access point each. The subset $\left\{w_{n^{I}}^{\mathrm{UL}}\right\}$ contains $N_{n^{I}}^{\mathrm{RU}}\cdot \left(N_{n^{I}}^{U}+N_{n^{I}}^{S}\right)$ one-dimensional routes and multi-dimensional routes formed on basis one-dimensional routes.

Since the robust method considers the worst case, the channel data rates in one-dimensional routes do not change depending on these routes’ links into multi-dimensional routes.

In [45], a method for estimating the channel data rate was developed. This work shows the dependence of the channel data rate on the signal reception algorithm $R^{S}$ and the signal-to-interference-plus-noise (SINR). According to this method, the channel data rates for one-dimensional routes can be defined as:

(7) $$V_{{\mathrm{gn}}_{n^{I}}^{U}\left(n_{n^{I}}^{S}\right)}^{\mathrm{DL}}\left(R^{S}\right)\leq 2\cdot V^{\mathrm{tech}}\cdot \overset{I^{O}}{\underset{i=1}{\sum }}\log_{2}\left(\frac{\sqrt{\rho_{{\mathrm{gin}}_{n^{I}}^{U}\left(n_{n^{I}}^{S}\right)}^{R^{S}\mathrm{DL}}}}{2\cdot {\mathrm{Erf}}^{-1}\left(P_{\max}^{\mathrm{Er}}/2\right)}+1\right),$$

(8) $$V_{{\mathrm{gn}}_{n^{I}}^{U}\left(n_{n^{I}}^{S}\right)}^{\mathrm{UL}}\left(R^{S}\right)\leq 2\cdot V^{\mathrm{tech}}\cdot \overset{I^{O}}{\underset{i=1}{\sum }}\log_{2}(\frac{\sqrt{\rho_{{\mathrm{gin}}_{n^{I}}^{U}\left(n_{n^{I}}^{S}\right)}^{R^{S}\mathrm{UL}}}}{2\cdot {\mathrm{Erf}}^{-1}\left(P_{\max}^{\mathrm{Er}}/2\right)}+1),$$

where $\rho_{{\mathrm{gin}}_{n^{I}}^{U}\left(n_{n^{I}}^{S}\right)}^{R^{S}\mathrm{DL}}$ is SINR in the downlink on the i-th subcarrier of the $n_{n^{I}}^{U}$-th user ($n_{n^{I}}^{S}$-th sensor) when transmitting data from the $n^{I}$-th access point along the route $w_{g^{*}}^{\mathrm{DL}}$. $\rho_{{\mathrm{gin}}_{n^{I}}^{U}\left(n_{n^{I}}^{S}\right)}^{R^{S}\mathrm{UL}}$ is SINR in the uplink on the i-th subcarrier of the $n^{I}$-th access point when transmitting data from the $n_{n^{I}}^{U}$-th user ($n_{n^{I}}^{S}$-th sensor) along the route $w_{g^{*}}^{\mathrm{UL}}$ when using the reception algorithm $R^{S}$. $V^{\mathrm{tech}}$ is the technical data rate, $I^{O}$ is the OFDM signal subcarriers’ quantity used for data transmission, $P_{\max}^{\mathrm{Er}}$ is the maximum allowable error probability for the transmitted data, and ${\mathrm{Erf}}^{-1}\left(●\right)$ is the inverse function of:

(9) $$\mathrm{Erf}\left(\rho \right)=\frac{1}{\sqrt{2\cdot \pi }}\overset{\infty }{\underset{\rho }{\int }}e^{-\frac{x^{2}}{2}}\mathrm{dx}.$$

In this case, it is sufficient to form one-dimensional routes’ subsets $\left\{w_{n^{I}}^{\mathrm{DL}}\right\}$ and $\left\{w_{n^{I}}^{\mathrm{UL}}\right\}$ at the analysis stage and multi-dimensional routes may be created at the routing stage during data transmission.

Therefore, using the robust method for estimating the channel data rate, the task of the routing stage is not to determine the optimal routes set, but the recurrent formation of multi-dimensional routes.

Consider the process of multi-dimensional routes forming in downlink. First, the user $n_{n^{I}}^{U}$ (sensor $n_{n^{I}}^{S}$) with a maximum current volume of delivered data $I_{n_{n^{I}}^{U}\left(n_{n^{I}}^{S}\right)}^{\mathrm{DL}}$ is found in the vector ${\vec{I}}^{\mathrm{DL}}$. Next, a one-dimensional route, $w_{{\mathrm{gn}}^{I}}^{\mathrm{DL}}$, that provides a maximum data rate to the user $n_{n^{I}}^{U}$ (sensor $n_{n^{I}}^{S}$) is selected from the subset $\left\{w_{n^{I}}^{\mathrm{DL}}\right\}$. The found one-dimensional route is appended to the multi-dimensional one, and the volume of data being delivered along this route, ${\tilde{I}}_{{\mathrm{gn}}_{n^{I}}^{U}\left(n_{n^{I}}^{S}\right)}^{\mathrm{DL}}$, is subtracted from the current volume of delivered data, $I_{n_{n^{I}}^{U}\left(n_{n^{I}}^{S}\right)}^{\mathrm{DL}}$, for the user $n_{n^{I}}^{U}$ (sensor $n_{n^{I}}^{S}$). Further, the procedure for appending one-dimensional routes is repeated until a multi-dimensional route of maximum dimension is formed, or until all accumulated data are transferred. A similar procedure works in the uplink.

**Figure 5:**
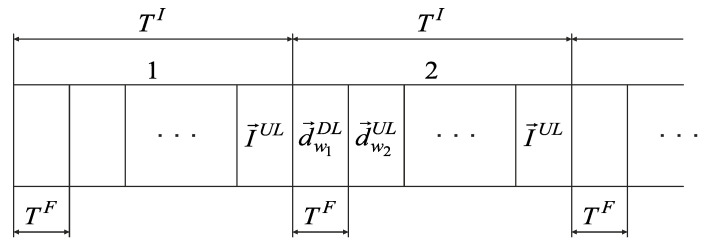
The data transmission structure in the CDR method using a robust method for estimating the channel data rate.

In this case, there is no need to transfer the optimal routes set ${\vec{N}}^{w\_\mathrm{opt}}$, and the data transmission structure in the CDR method takes the form shown in Figure 5.

When using the robust method, users, sensors, and BR also accumulate the received data on the interval $T^{I}$. BR requests the data volumes ${\vec{I}}^{\mathrm{UL}}$ accumulated by devices from access points in the last frame $T^{F}$ of each interval $T^{I}$. Further, in each frame $T^{F}$ of all intervals $T^{I}$, except the first, BR builds a multi-dimensional route using the accumulated ${\vec{I}}^{\mathrm{DL}}$ and the received ${\vec{I}}^{\mathrm{UL}}$ vectors.

In the case of data transmission from sensors, the CDR method with a robust method for estimating the channel data rate has the following disadvantages:

- Since the optimal routes set ${\vec{N}}^{w\_\mathrm{opt}}$ is not formed, there is no information about the resource allocation for each sensor. In this case, all sensors’ receivers must be constantly switched on, increasing their power consumption.
- Transmitting the data volumes ${\vec{I}}^{\mathrm{UL}}$ from all sensors in each interval $T^{I}$ leads to an increase in the service data volume.
- The constant frame duration $T^{F}$ when transmitting a small data volume from sensors is inefficient using channels.

In this regard, it is necessary to develop a novel modification of the CDR method. It should include the following changes:

- Increase the sensor polling period to $T^{S}$. This interval is equal to the certain number of data accumulation intervals $T^{I}$. It could be reached by splitting the sensors into $N^{\mathrm{SP}}$ groups, where each group $n^{\mathrm{SP}}=\bar{1,N^{\mathrm{SP}}}$ is serviced on interval $T^{I}$.
- Split the current volumes of delivered data ${\vec{I}}^{\mathrm{DL}}$ and ${\vec{I}}^{\mathrm{UL}}$ on ${\vec{I}}^{\mathrm{DU}}$ and ${\vec{I}}^{\mathrm{UU}}$ for users, and ${\vec{I}}^{\mathrm{DS}}$ and ${\vec{I}}^{\mathrm{US}}$ for sensors.
- Split the data accumulation interval $T^{I}$ into two parts with different frame durations $T^{\mathrm{FU}}$ for users and $T^{\mathrm{FS}}$ for sensors.
- Construct a one-dimensional routes vector $\vec{w}$ per BR for each frame $T^{\mathrm{FS}}$, which contains one-dimensional routes to deliver data to or from sensors.

### 3.4. Novel Modification of the CDR Method

**Figure 6:**
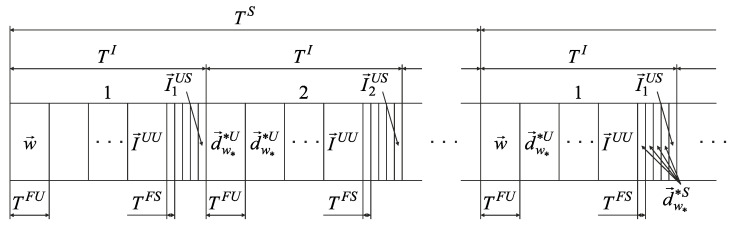
Data transmission structure for novel modification of the CDR method.

In the novel modification of the CDR method, users and sensors are serviced at interval $T^{I}$ in turn, with different frame durations, $T^{\mathrm{FU}}$ and $T^{\mathrm{FS}}$, respectively. Therefore, four groups of one-dimensional routes’ subsets are determined at the analysis stage: $\left\{w_{n^{I}}^{\mathrm{DL}\_U}\right\}$, $\left\{w_{n^{I}}^{\mathrm{UL}\_U}\right\}$ for users and $\left\{w_{n^{I}}^{\mathrm{DL}\_S}\right\}$, $\left\{w_{n^{I}}^{\mathrm{UL}\_S}\right\}$ for sensors. According to the robust method of estimating the channel data rates, $V_{{\mathrm{gn}}_{n^{I}}^{U}\left(n_{n^{I}}^{S}\right)}^{\mathrm{DL}\left(\mathrm{UL}\right)}\left(R^{s}\right)$ is determined for the worst-case scenario for each one-dimensional route using expressions (7) and (8). For each route, the data volumes, ${\tilde{I}}_{{\mathrm{gn}}_{n^{I}}^{U}}^{\mathrm{DL}\left(\mathrm{UL}\right)}$, delivered to (from) the user $n_{n^{I}}^{U}$ for the frame $T^{\mathrm{FU}}$ duration and the volumes of data being delivered, ${\tilde{I}}_{{\mathrm{gn}}_{n^{I}}^{S}}^{\mathrm{DL}\left(\mathrm{UL}\right)}$, to (from) the sensor $n_{n^{I}}^{S}$ for the frame $T^{\mathrm{FS}}$ duration are calculated. Further, a certain quantity of frame durations $T^{\mathrm{FS}}$ are reserved on the data vector accumulation interval $T^{I}$ based on the known sensors’ quantity in the group, the maximum transmitted data volumes, and the calculated delivered data volumes. In this case, the data transmission structure of the novel modification of the CDR method takes the form shown in Figure 6.

Since the changes do not concern data transmission between BR and users, only the case of data transmission with sensors will be considered.

In the beginning of the first period $T^{S}$, the BR does not have information about the data volumes ${\vec{I}}^{\mathrm{DS}}$ and ${\vec{I}}^{\mathrm{US}}$. The BR sends the one-dimensional routes’ vector $\vec{w}$ to all access points in the first frame $T^{\mathrm{FU}}$ of this period $T^{S}$. The route vector $\vec{w}$ provides for the transfer-only data volumes ${\vec{I}}^{\mathrm{US}}$ from each sensor’s group on the last frame $T^{\mathrm{FS}}$ of each interval $T^{I}$. Starting from the second interval $T^{I}$, the one-dimensional routes’ vector $\vec{w}$ is calculated on BR taking into account the vectors ${\vec{I}}^{\mathrm{DS}}$ and ${\vec{I}}^{\mathrm{US}}$. However, these data are not transmitted immediately, and the formed one-dimensional routes for each frame are appended to the one-dimensional routes’ vector $\vec{w}$. From the second period $T^{S}$ in the first frame $T^{\mathrm{FU}}$, a calculated one-dimensional routes’ vector $\vec{w}$ is sent to all access points and the accumulated data are transmitted according to the constructed routes.

## 4. Evaluation of Novel Modification of Collective Dynamic Routing Method Effectiveness

The evaluation of the novel modification of the CDR method’s effectiveness was provided by modeling a seamless IEEE 802.11ax network segment.

In the novel modification of the CDR method, only the routing stage is changed. Therefore, the simulation was carried out with the constant and known traffic and communication channels’ parameters. Rotes reconfiguration in dynamic routing protocols is only used in the case of changing the topology or traffic parameters. In this scenario, the routes do not vary, and any dynamic routing protocol becomes a static one.

**Figure 7:**
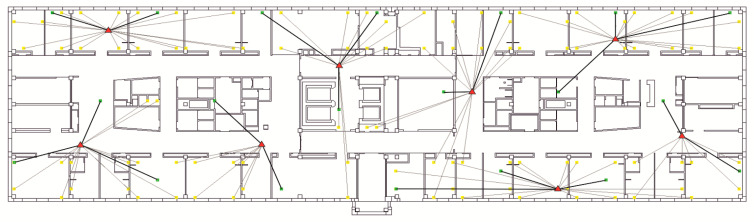
Access points, users, and sensors’ location of the seamless Wi-Fi network segment.

The Wi-Fi network segment consists of 8 Eltex WEP-3ax access points working on 1, 6, and 11 frequency channels in the 2.4 GHz frequency range, with a 20 MHz band. The network is designed to serve 20 users and 100 stationary sensors. The location of access points, users, and sensors is shown in Figure 7.

The locations of access points are indicated by red triangles, and users and sensors by green and yellow dots, respectively.

The simulation was provided using the “OFDM Planning” software package. SINR’s for users and sensors were calculated on the basis of the floor plan, according to the International Telecommunication Union recommendations ITU-R R.1238-10 [46] and ITU-R R.2040-1 [47].

Each device connects to an access point that provides the highest channel data rate among available access points. In accordance with the obtained network topology, one-dimensional routes’ subsets $\left\{w_{n^{I}}^{\mathrm{DL}\_U}\right\}$, $\left\{w_{n^{I}}^{\mathrm{UL}\_U}\right\}$, $\left\{w_{n^{I}}^{\mathrm{DL}\_S}\right\}$, and $\left\{w_{n^{I}}^{\mathrm{UL}\_S}\right\}$ are formed on each access point. Such routes provide the maximum data rate for each device.

In this case, considering the absence of service traffic in static routing, this type of routing will always have better characteristics than any dynamic protocol. Therefore, the effectiveness comparison is provided only for the novel modification of the CDR method and static routing.

The connection of users and sensors to access points is indicated in Figure 7 by bold and thin black lines, respectively.

In the simulation, TCP, HTTP, and FTP protocol packets are generated using the 4IPP model [48]. The traffic transfer rates $V^{T}$ for all users were the same and varied in the range from 1 to 10 Mbps. The information vector accumulation interval $T^{I}$ also varied in the range from 10 to 100 ms. The packets transmitted by the sensors were generated according to the Pareto model [49], and the average volume of data delivered to the sensors was 100 bytes, and from the sensors 1 kB per second.

Traffic transmission over a seamless IEEE 802.11ax network segment is simulated as follows.

Initially, the “OFDM Planning” software package generates packets in accordance with the specified models, indicating the arrival time and the length of each packet.

Further, the generated packets are transmitted over the network using both novel modification of the CDR method and static routing.

When using the novel modification of the CDR method, the entire simulation time is divided into $T^{I}$ duration intervals. Further, for each interval $T^{I}$, packets of all users and the served sensors group received during its duration are selected. Based on this information, vectors ${\vec{I}}^{\mathrm{DU}}$, ${\vec{I}}^{\mathrm{UU}}$, ${\vec{I}}^{\mathrm{DS}}$, and ${\vec{I}}^{\mathrm{US}}$ are formed. Multi-dimensional routes are formed according to the algorithm in [45] and described in the article when considering a robust method for estimating the channel data rate.

When using static routing, the users’ and sensors’ data are transmitted in $T^{\mathrm{FU}}$ duration frames. Therefore, multi-dimensional routes are formed on each frame using packets that have arrived before the end of this frame in the order of their arrival time. Multi-dimensional routes for static routing are formed by the following algorithm.

Initially, the first packet not delivered to (from) the user (sensor) is found. Next, a one-dimensional route is selected from the valid routes set {w} that provides the maximum data transfer rate from (to) this user (sensor). The found one-dimensional route appends to the multi-dimensional one. The procedure for appending one-dimensional routes is repeated until a multi-dimensional maximum dimension route is formed. For the found multi-dimensional route, the data rates considering the intra-system interference influence and the volumes of data delivered are calculated. Packets whose entire data have been delivered are removed, and the length of partially delivered packets is reduced by the volumes of data delivered. Further, the formation of routes continues until all packets are transmitted.

Based on the simulation results, the load on the network $A^{\mathrm{net}}$, the data transfer rate over the network as a whole for users $V^{\Sigma U}$ and sensors $V^{\Sigma S}$, and the ratios of the average active operation time of the sensor’s receiver and transmitter to the sensor polling period $A^{\mathrm{RS}}$ and $A^{\mathrm{TS}}$, respectively, were calculated.

When simulating a novel modification of the CDR method, the sensors were separated into 10–100 groups depending on the information accumulation vector interval $T^{I}$ and the sensor polling period $T^{S}$. From 1 to 10 sensors were serviced at each interval $T^{I}$. The data transmission frame duration to or from users was $T^{\mathrm{FU}}$ = 720.8 μs and the data transmission frame duration to or from sensors was $T^{\mathrm{FS}}$ = 190.4 μs. The radiated power for access points and users was considered equal to 19 dBm, and 0 dBm for sensors.

The load on the network $A^{\mathrm{net}}$ characterizes the existing network capacity margin and is defined as the ratio of the time occupied by data transmission from (to) devices to the sensor polling period $T^{S}$. For the novel modification of the CDR method, the network load can be defined as:

(10) $$A^{net}=\frac{1}{T^{S}}\cdot \left(T^{FU}\cdot \left(\sum_{n^{SP}=1}^{N^{SP}}N_{n^{SP}}^{FU}+1\right)+T^{FS}\cdot \sum_{n^{SP}=1}^{N^{SP}}N_{n^{SP}}^{FS}\right),$$

where $N_{n^{\mathrm{SP}}}^{\mathrm{FU}},$ is the frame quantity during which data were transmitted to (from) users, and $N_{n^{\mathrm{SP}}}^{\mathrm{FS}}$ is the frame quantity during which data were transmitted to (from) sensors at the $n^{\mathrm{SP}}$-th information accumulation interval $T^{I}$.

The ratios $A^{\mathrm{RS}}$ and $A^{\mathrm{TS}}$ show the parts of the sensor’s receiving and transmitting device activity time, which, along with the magnitude of its emitted power, significantly affects the wireless sensor lifetime.

**Figure 8:**
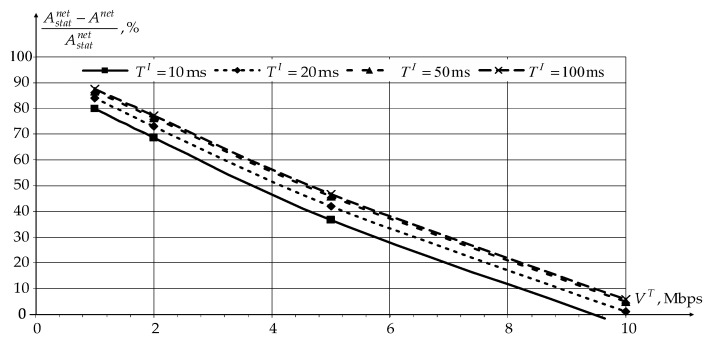
The dependencies of reducing the network load $A^{\mathrm{net}}$ on the traffic transfer rate $V^{T}$ by users for different intervals $T^{I}$.

**Figure 9:**
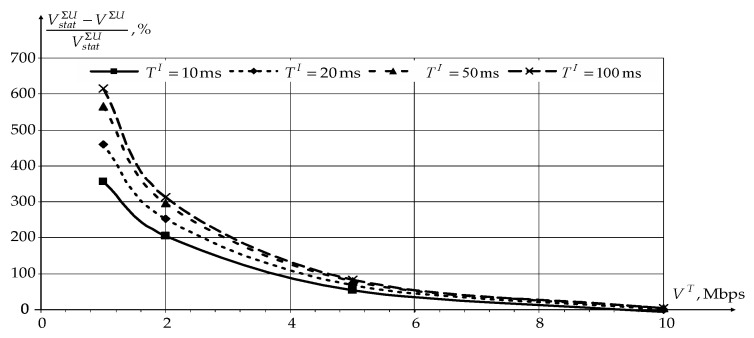
The dependencies of the increase in the data transfer rate over the network for all users $V^{\Sigma U}$ for different intervals $T^{I}$.

Since in the novel modification of the CDR method, users and sensors are serviced in the interval $T^{I}$ in turn, its effectiveness analysis for users was first carried out. The analysis was carried out depending on the duration $T^{I}$ and the user’s traffic transfer rate $V^{T}$ at $T^{S}$= 1s. Figure 8 and Figure 9 show the dependencies of reducing the network load $A^{\mathrm{net}}$ and increasing the data transfer rate over the network $V^{\Sigma U}$ for users using novel modification of the CDR method compared to static routing ($A_{\mathrm{stat}}^{\mathrm{net}}$ and $V_{\mathrm{stat}}^{\Sigma U}$).

According to Figure 8 and Figure 9, application of the novel modification of the CDR method reduces the network load $A^{\mathrm{net}}$ and increases the data transfer rate over the network $V^{\Sigma U}$ for users in general. At the same time, the novel modification of the CDR method’s effectiveness decreases with an increase in the traffic transfer rate by users. When the network is fully loaded, static routing even has a slight gain compared to the novel modification of the CDR method, since static routing does not need to transmit service information.

An increase in the information accumulation interval $T^{I}$ also contributes to an increase in novel modification of the CDR method’s effectiveness. However, when $T^{I}$ becomes greater than 50 ms, there is no further increase in the efficiency. At the same time, the use of an information accumulation interval of more than 20 ms may have negative consequences for time-dependent traffic. Therefore, for further investigation, the information accumulation interval duration is chosen as 20 ms. In this case, the novel modification of the CDR method’s efficiency will decrease by no more than 5%.

The main indicators of the novel modification of the CDR method’s effectiveness for sensors are the total data transfer rate over the network $V^{\Sigma S}$ and the ratios $A^{\mathrm{RS}}$ and $A^{\mathrm{TS}}$. The packet length of sensors’ traffic and the number of generated packets per time unit are usually constant. In this case, the novel modification of the CDR method’s effectiveness is analyzed depending on the sensors’ polling period $T^{S}$ only. The values for the polling period $T^{S}$ vary in the range from 0.2 to 2 s.

**Figure 10:**
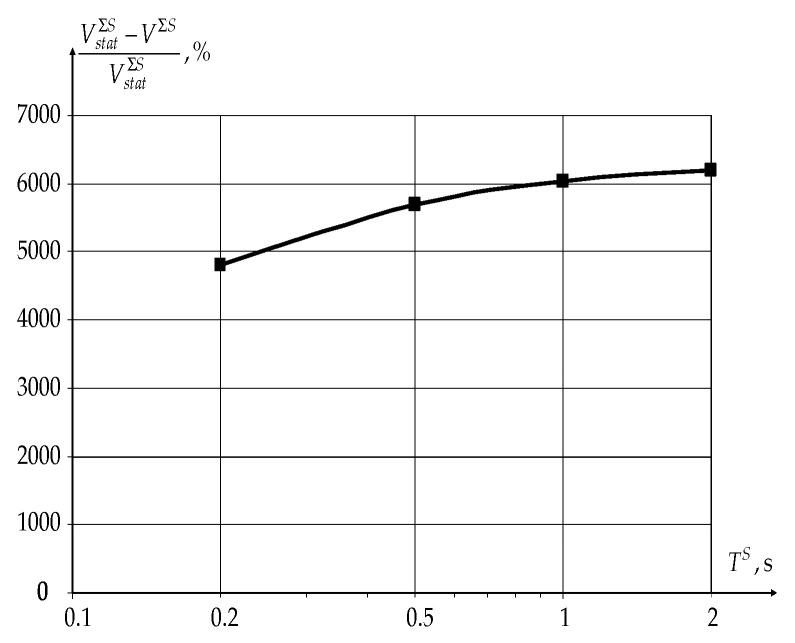
The dependence of the increasing data transfer rate over the network as a whole $V^{\Sigma S}$ on the sensor polling period $T^{S}$.

Figure 10 shows the dependence of the increasing data transfer rate over the network $V^{\Sigma S}$ for sensors using the novel modification of the CDR method on the sensor polling period compared to static routing ($V_{\mathrm{stat}}^{\Sigma S}$).

The use of the novel modification of the CDR method allows to increase the data transfer rate over the network as a whole for sensors by more than 50 times according to the graph in Figure 10. At the same time, the novel modification of the CDR method’s effectiveness grows with an increasing sensor polling period $T^{S}$ as well as for the case of data transmission by users.

The closest case was considered in [28]. However, the simulation in this work was carried out for the case of using 996 subcarriers (80 MHz bandwidth) and 4×1 MISO. Under these conditions, for the case considered in the article, the total radiated power, taking into account airtime, will be 79 mW. At the same time, the network throughput is approximately 2.8 Gbps (Figure 5 in [28]). For the novel modification of the CDR method, in this case, the network throughput will be 5.6 Gbps, with a large service area.

**Figure 11:**
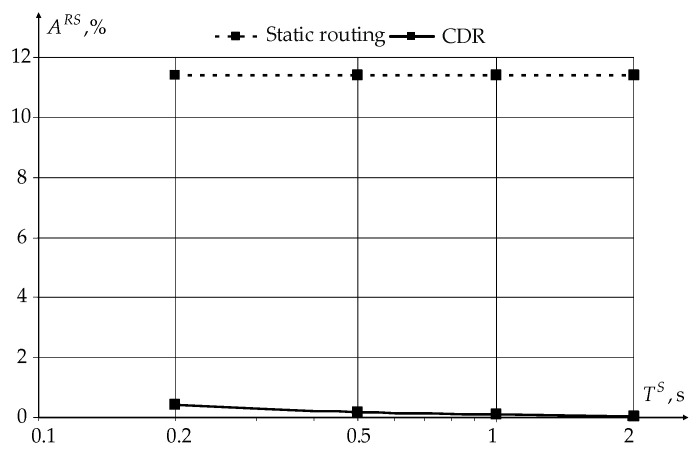
Dependencies of the ratio $A^{\mathrm{RS}}$ of the average sensor receiver active time to the sensor polling period $T^{S}$.

**Figure 12:**
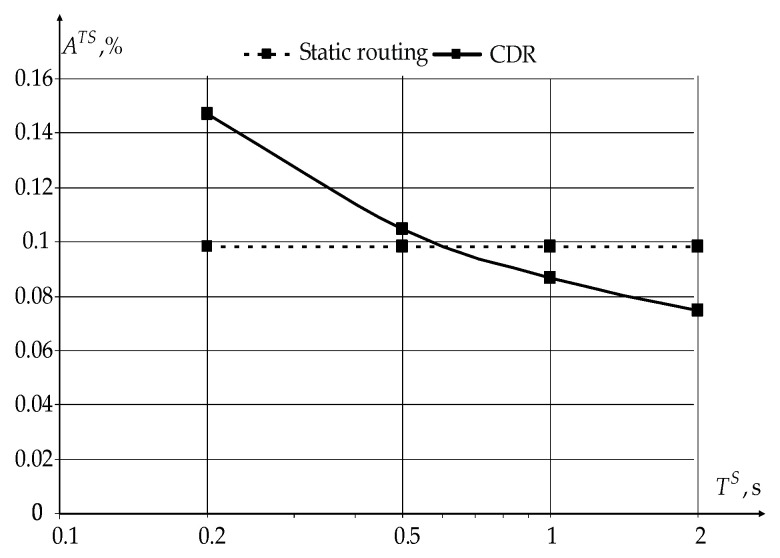
Dependencies of the ratio $A^{\mathrm{TS}}$ of the average sensor transmitter active time to the sensor polling period $T^{S}$.

Figure 11 and Figure 12 show the dependencies of the ratio of the sensor’s receiver $A^{\mathrm{RS}}$ and transmitter $A^{\mathrm{TS}}$ average active times to the sensor polling period $T^{S}$.

From the graphs shown in Figure 11 and Figure 12, it can be seen that the use of the novel modification of the CDR method reduces the sensor receiver’s active time from 12% (for static routing) to less than 1%, with almost the same transmitter operating time. The activity time of both receivers and transmitters slowly decreases with the increasing sensors’ polling period.

The average of the wireless sensor current consumption $I^{\mathrm{SEN}}$ consists of three components: the current consumption of the sensor’s processor $I^{\mathrm{CPU}}$ and the current consumption averaged over the polling period by the radio module in the receiving (listening) $I^{\mathrm{RS}}$ and transmitting $I^{\mathrm{TS}}$ modes:

(11) $$I^{\mathrm{SEN}}=I^{\mathrm{CPU}}+A^{\mathrm{RS}}\cdot I^{\mathrm{RS}}+A^{\mathrm{TS}}\cdot I^{\mathrm{TS}},$$

Consider a wireless sensor built around an ESP32 microcontroller with a Wi-Fi module that has $I^{\mathrm{CPU}}$ = 3 mA, $I^{\mathrm{RS}}$ = 80 mA, and $I^{\mathrm{TS}}$ = 120 mA at an output power of 0 dBm [50]. The sensor current consumption when using the novel modification of the CDR method is reduced from 12 to 3.5 mA. Therefore, when using a new modification of the CDR method, the main energy consumer in the sensor becomes its processor, and not the Wi-Fi module, the average consumption of which is 6 times less than the processor.

Thus, the novel modification of the CDR method for IEEE 802.11ax allows reducing the sensor’s active operation time and the wireless sensor’s power consumption. At the same time, this method provides a high transfer rate and a short data delivery time for users.

## 5. Conclusions

This paper described the development of a novel modification of the collective dynamic routing method that allows public wireless networks to simultaneously serve both users and sensors for sensors’ communication in wireless public networks. For the chosen seamless IEEE 802.11ax network segment, the effectiveness of using the novel modification of the collective dynamic routing method was demonstrated.

The novel modification of the collective dynamic routing method improved the data rate for sensors by more than 50 times for the considered network. Additionally, the operation time for the sensor’s receiver became less than 1% of the polling period with the same quality of service for the users. This reduced the wireless sensor’s average current consumption radio modules based on the ESP32 microcontroller by 18 times.
